# Supplementary material for: Indirect Benefits of Seasonal Malaria Chemoprevention for Non-Malarial Pediatric Infections and Routine Antibiotic Use in Real-World Programmatic Settings: A Pre-Post Study Using Positive and Negative Controls
Source: medRxiv. 2025 May 9:2025.05.08.25327228. Preprint. [Version 1] doi: 10.1101/2025.05.08.25327228 (PMC12248168; doi:10.1101/2025.05.08.25327228)
Supplement: Supplement 1 — Supplementary Table 1- Changes in Diagnoses and Treatment rates following SMC by Month of SMC Administration and Overall (IRR, 95% CI) Supplementary Table 2- Incidence Rates, Rate Ratios, and Rate Differences of Outcomes During SMC Administration Weeks and the First, Second-, and Third-Weeks Post-Administration Supplementary Figure 1- Diagnoses Rates and Treatment Prescription Rates for Control Outcomes in 2020 and 2021 in the Context of SMC Administration Supplementary Figure 2- Difference in Incidence Rates of Diagnoses and Treatment During SMC Administration vs. Post-Administration Weeks, by Month of Administration for Control Outcomes [file NIHPP2025.05.08.25327228v1-supplement-1.pdf]

Supplementary Table 1- Changes in Diagnoses and Treatment rates following SMC by Month of SMC Administration and Overall (IRR, 95% CI)

Outcome	Overall	July	August	September	October	P value for global test of interaction
Pneumonia	0.86 (0.79 to 0.93)	0.66 (0.57 to 0.74)	0.92 (0.80 to 1.05)	1.0 (0.83 to 1.18)	0.81 (0.69 to 0.92)	0.000
Diarrhea	0.83 (0.74 to 0.93)	1.02 (0.82 to 1.22)	0.74 (0.61 to 0.88)	0.85 (0.70 to 1.0)	0.82 (0.67 to 0.97)	0.047
Acute malnutrition	0.71 (0.51 to 0.97)	0.49 (0.03 to 0.94)	0.69 (0.23 to 1.14)	0.81 (0.29 to 1.34)	0.80 (0.47 to 1.13)	0.654
Malaria (positive control)	0.62 (0.56 to 0.69)	0.90 (0.73 to 1.07)	0.59 (0.52 to 0.67)	0.54 (0.47 to 0.61)	0.67 (0.58 to 0.75)	0.000
Injury (negative control)	0.89 (0.67 to 1.19)	0.92 (0.46 to 1.37)	1.03 (0.57 to 1.48)	0.80 (0.44 to 1.16)	0.82 (0.43 to 1.20)	0.861
Antibiotic prescription	0.88 (0.83 to 0.94)	0.78 (0.70 to 0.86)	0.92 (0.84 to 1.0)	1.0 (0.86 to 1.13)	0.81 (0.72 to 0.91)	0.002
Antimalarial prescription (positive control)	0.63 (0.56 to 0.69)	0.90 (0.72 to 1.07)	0.60 (0.53 to 0.68)	0.54 (0.47 to 0.61)	0.67 (0.58 to 0.76)	0.000

medRxiv preprint doi: https://doi.org/10.1101/2025.05.08.25327228; this version posted May 9, 2025. The copyright holder for this preprint (which was not certified by peer review) is the author/funder, who has granted medRxiv a license to display the preprint in perpetuity.
It is made available under a CC-BY 4.0 International license .

Supplementary Table 2- Incidence Rates, Rate Ratios, and Rate Differences of Outcomes During SMC Administration Weeks and the First, Second-, and Third-Weeks Post-Administration

**Weeks post SMC administration**	**Incidence rate per 1,000 person-week (95%CI)**	**Incidence rate ratio (95%CI)**	**Incidence Rate Difference per 1,000 person-week (95%CI)**
Malaria (positive control)			
Admin week	9.7 (8 to 11.4)	Ref	Ref
1	6 (4.9 to 7.1)	0.61 (0.55 to 0.68)	-3.8 (-4.8 to -2.8)
2	5.8 (4.9 to 6.8)	0.6 (0.53 to 0.68)	-3.9 (-5.1 to -2.7)
3	6.4 (5.4 to 7.3)	0.65 (0.59 to 0.73)	-3.4 (-4.5 to -2.3)
Injury (negative control)			
Admin week	0.17 (0.12 to 0.23)	Ref	Ref
1	0.14 (0.11 to 0.17)	0.81 (0.58 to 1.13)	-0.03 (-0.09 to 0.02)
2	0.17 (0.13 to 0.21)	0.98 (0.73 to 1.31)	0.0 (-0.06 to 0.05)
3	0.15 (0.11 to 0.2)	0.89 (0.63 to 1.26)	-0.02 (-0.08 to 0.04)
Pneumonia			
Admin week	4.7 (3.8 to 5.7)	Ref	Ref
1	4.3 (3.5 to 5)	0.9 (0.83 to 0.97)	-0.5 (-0.9 to -0.1)
2	4.1 (3.2 to 4.9)	0.86 (0.78 to 0.95)	-0.7 (-1.1 to -0.2)
3	3.9 (3.1 to 4.6)	0.82 (0.74 to 0.90)	-0.9 (-1.3 to -0.4)
Diarrhea			
Admin week	1.4 (1 to 1.7)	Ref	Ref
1	1.2 (0.9 to 1.5)	0.87 (0.77 to 0.98)	-0.2 (-0.3 to 0)
2	1 (0.8 to 1.3)	0.77 (0.66 to 0.90)	-0.3 (-0.5 to -0.1)
3	1.2 (0.9 to 1.5)	0.86 (0.76 to 0.97)	-0.2 (-0.3 to 0)
Acute Malnutrition			
Admin week	0.17 (0.09 to 0.24)	Ref	Ref
1	0.12 (0.05 to 0.2)	0.74 (0.49 to 1.11)	-0.04 (-0.1 to 0.01)
2	0.1 (0.04 to 0.17)	0.63 (0.42 to 0.94)	-0.06 (-0.11 to -0.01)
3	0.13 (0.06 to 0.19)	0.76 (0.54 to 1.06)	-0.04 (-0.09 to 0.01)
Antimalarial prescription (positive control)			
Admin week	9.7 (7.9 to 11.4)	Ref	Ref
1	6 (4.8 to 7.1)	0.62 (0.56 to 0.69)	-3.7 (-4.7 to -2.7)
2	5.8 (4.8 to 6.8)	0.60 (0.53 to 0.68)	-3.9 (-5.1 to -2.7)
3	6.4 (5.4 to 7.4)	0.66 (0.59 to 0.74)	-3.3 (-4.4 to -2.2)
Antibiotic prescription			
Admin week	7.9 (6.6 to 9.2)	Ref	Ref
1	7.1 (5.8 to 8.3)	0.90 (0.84 to 0.96)	-0.82 (-1.33 to -0.31)
2	6.9 (5.7 to 8)	0.87 (0.81 to 0.93)	-1.02 (-1.57 to -0.46)
3	6.9 (5.7 to 8.1)	0.87 (0.8 to 0.95)	-0.99 (-1.61 to -0.36)

medRxiv preprint doi: https://doi.org/10.1101/2025.05.08.25327228; this version posted May 9, 2025. The copyright holder for this preprint (which was not certified by peer review) is the author/funder, who has granted medRxiv a license to display the preprint in perpetuity.
It is made available under a CC-BY 4.0 International license .

Supplementary Figure 1- Diagnoses Rates and Treatment Prescription Rates for Control Outcomes in 2020 and 2021 in the Context of SMC Administration

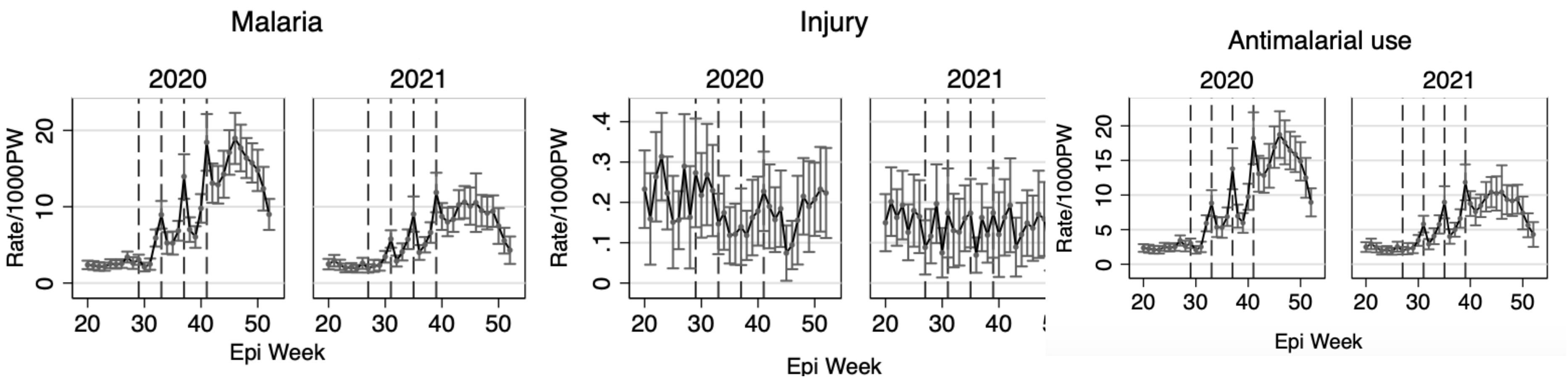

medRxiv preprint doi: https://doi.org/10.1101/2025.05.08.25327228; this version posted May 9, 2025. The copyright holder for this preprint (which was not certified by peer review) is the author/funder, who has granted medRxiv a license to display the preprint in perpetuity.
It is made available under a CC-BY 4.0 International license .

Supplementary Figure 2- Difference in Incidence Rates of Diagnoses and Treatments During SMC Administration vs. Post-Administration Weeks, by Month of Administration for Control Outcomes

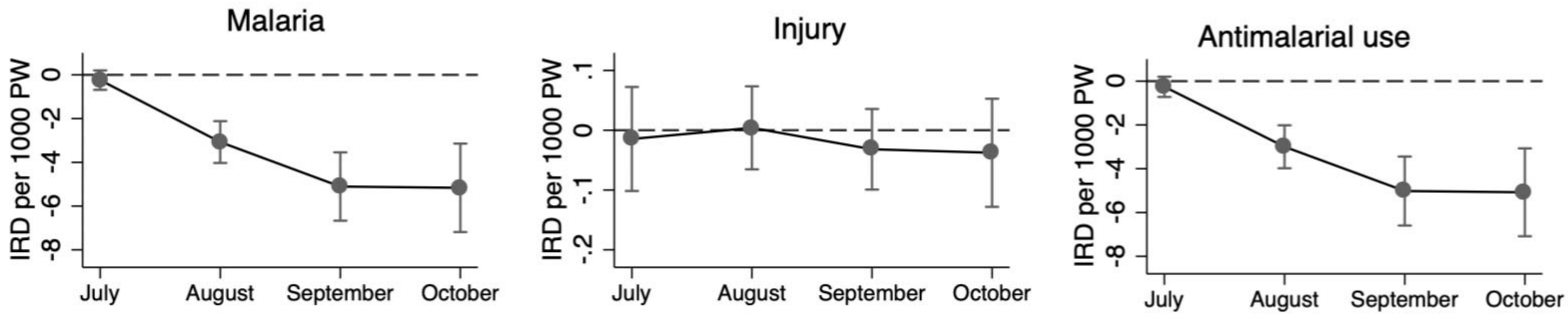

Note: P-values from the global test of interaction between SMC and month of administration were 0.000 and 0.861 for malaria and injury, respectively, and 0.001 for antimalarial prescription rates.

medRxiv preprint doi: https://doi.org/10.1101/2025.05.08.25327228; this version posted May 9, 2025. The copyright holder for this preprint (which was not certified by peer review) is the author/funder, who has granted medRxiv a license to display the preprint in perpetuity.
It is made available under a CC-BY 4.0 International license .
